# The influencing factors, diversification trends, and motivation-enhancement strategies of learning motivation among undergraduate students in Chinese application-oriented universities in the digital age: a grounded theory analysis based on Y University

**DOI:** 10.3389/fpsyg.2026.1902017

**Published:** 2026-09-01

**Authors:** Menglu Guo, Qiaoxia Wang, Li Zhang, Linzhi He

**Affiliations:** 1 School of Accounting, Wuchang Institute of Technology, Wuhan, China; 2 School of Education, Central China Normal University, Wuhan, China

**Keywords:** application-oriented university, digital and intelligent era, grounded theory, learning motivation, self-efficacy

## Abstract

Artificial intelligence technology is accelerating the transformation of the teaching ecosystem in universities, and as a result, the learning motivation of college students is profoundly affected. To reveal the core influencing factors and mechanism of learning motivation for Chinese application-oriented university students in the digital era, using grounded theory, 30 undergraduate students from Y University were selected as the research subjects. Through semi-structured interviews, original data were collected. Through open coding, axial coding, and selective coding, concepts and categories were refined, and a theoretical saturation test was conducted. The study found that the learning motivation of undergraduate students in application-oriented universities in the digital era is driven by four main categories: the core driving layer (AI-specific dimension, professional cognition and expectations, self-awareness, self-achievement, internal drive), the basic guarantee layer (school resource conditions, course evaluation methods), the relationship influence layer (teacher and peer influence, family influence), and the external constraint layer (student behavior constraints, autonomous time allocation). It presents a diversified trend and ultimately forms a “cognition-context” dual-drive influence mechanism model. Based on this, from the aspects of internal cognitive stimulation, external condition improvement, interpersonal relationship drive, and learning situation creation, practical paths are provided for students in application-oriented universities to adapt to the development trend of the digital era and stimulate their learning motivation.

## 1 Introduction

With the advancement of society and education, learning motivation continues to evolve and is influenced by individual differences in their perception of society, thereby exhibiting complexity and diversity (Shi, 2011). Currently, the rapid development of artificial intelligence technology is transforming education; some students experience weakened motivation and a tendency toward utilitarian learning due to factors such as “AI-induced career substitution anxiety”; others rely heavily on AI tools during learning, leading to excessive dependence that undermines their autonomy in learning.

Atkinson and Litwin (1960) first categorized learning motivation into intrinsic motivation (e.g., curiosity, interest, thirst for knowledge) and extrinsic motivation (e.g., awards, employment, further education), and constructed achievement motivation as a two-dimensional model comprising “motivation to achieve success” and “motivation to avoid failure” (Atkinson and Litwin, 1960). Keirsey and Bates (1984) noted that “everyone is driven by instincts and pursues them as goals, with each individual identifying one instinct as paramount”. Bandura (1986) termed the interplay of individual factors such as behavior and cognition with environmental influences as the “triangular interaction”. Ausubel was the first to elucidate the multifaceted nature of learning motivation, proposing that students’ classroom motivation consists of three components: cognitive drive, self-improvement drive, and affiliation drive. Cognitive drive stems from curiosity and the desire to explore knowledge itself; self-improvement drive aims to enhance status and self-esteem through academic achievement; affiliation drive seeks recognition and approval from elders (Ausubel, 1994).

Lu et al. (1992) identified three key characteristics of college students’learning motivation: diversity, dynamism, and dualistic nature. They also proposed five strategies to enhance learning motivation: providing life philosophy education; setting clear learning objectives; creating problem-based scenarios to stimulate new intellectual curiosity; emphasizing feedback during learning processes; and appropriately introducing competitive mechanisms. Mao (1995) highlighted four features of college students’ learning motivation: complex and highly unstable motivations coupled with unclear goals; a predominant focus on immediate and direct outcomes; utilitarian and practical motivations; and moderate levels of achievement motivation. Liu and Xiong (2003) outlined five approaches to fostering learning motivation: cultivating proper life perspectives, enhancing cognitive abilities, stimulating interest in learning, transforming personal motivations into social-driven drives, and helping students recognize learning as essential for self-improvement and personal development. Research by Chi et al. (Chi and Xin, 2006) revealed that students with high self-efficacy exhibit strong confidence and intrinsic learning motivation, being less influenced by external factors.

Based on current research, the field of learning motivation has developed a theoretical system centered on the Self-Determination Theory and the Self-Efficacy Theory. However, there are two limitations in related research: Firstly, the existing studies on the relationship between artificial intelligence and college students’ learning motivation mostly select students from public comprehensive universities as the research sample, and rarely focus on the application-oriented university group. Application-oriented universities focus on vocational training and practical job skills acquisition as their core training orientation. The AI career substitution anxiety, access to digital learning resources, and utilitarian learning demands faced by students in application-oriented universities are significantly different from those in public universities. The existing results cannot fully explain the unique logic of the formation of this group’s learning motivation; Secondly, current research on digital education mostly focuses on the development of AI teaching tools and the optimization of online classroom technologies, lacking qualitative in-depth exploration from the perspective of students’ subjective psychology. Many quantitative studies only verify the correlation of a single variable, lacking a grounded theory that self-organizes from the bottom up to refine a localized motivation model, and failing to fully capture the multi-layer influence paths of AI over-reliance, digital self-efficacy, and other psychological variables specific to the new era on learning motivation. The existing research has not yet established a complete framework for the influence mechanism of motivation in China’s application-oriented university students in line with the digital age.

Based on this, this study is based on the background of the digital and intelligent era and takes the college students of Y University as the research object. It adopts the grounded theory method to explore the following questions: (1) What are the core influencing factors of the learning motivation of college students in application-oriented universities in the digital and intelligent era? (2) Through what mechanisms do each of these influencing factors affect the generation and maintenance of learning motivation? (3) How can learning motivation stimulation strategies be proposed based on these influencing mechanisms?

This study clearly sets three core research objectives: First, systematically extract all the influencing factors of the learning motivation of undergraduate students in applied universities in the digital intelligence environment, including digital-specific variables such as the use of AI tools and digital career anxiety; Second, clarify the interaction paths between internal cognition and external learning contexts, and build a “cognition-context” dual-driven mechanism model for the influence of learning motivation; Third, based on the characteristics of the educational resources of applied universities, propose practical strategies for stimulating students’ learning motivation that are suitable for the teaching scenarios of the AI era and can be implemented.

Based on these research objectives, this paper focuses on answering three core research questions: (1) what are the core influencing factors of the learning motivation of undergraduate students in applied universities in the digital intelligence era? (2) Through what internal logic do various influencing factors jointly drive the diversified shift of students’ learning motivation? (3) In combination with the digital intelligence teaching environment, how to formulate a learning motivation stimulation path that is suitable for applied universities?

## 2 Research process

### 2.1 Research methods and data collection

The sample selected for this study, Y University, is an applied-type university. The undergraduate students currently enrolled were randomly chosen as the research subjects. The number of interviewees was determined based on the principle of theoretical saturation. In this study, a total of 30 students participated in the in-depth interviews, including 7 freshmen, 9 sophomores, 9 juniors, and 5 seniors, covering majors such as Business Administration, Accounting, Calligraphy, Photography, Digital Media Art, Communication Engineering, Electrical Engineering, and Mathematics. To enable the respondents to present the interview content more realistically and accurately, a 5-min discussion on the types of learning motivation would be conducted before the formal interview. To prevent preconceived notions about the students, a semi-structured interview was adopted, with the interview outline centered around “Learning Motivation in the Digital Age.” To deeply explore the influencing factors of learning motivation in the digital age, the research team compiled the interview outline as follows: (1) “What do you think are the main motivations for your learning in the AI era?” (2) “How do AI technologies (such as AI tools, AI teaching content) affect your learning motivation?” (3) “Which external environmental factors (such as course design, school support) will enhance or weaken your learning motivation?” (4) “Please share an experience where your learning motivation was particularly strong or particularly weak.” (5) “How do you think the AI era has influenced factors such as cognition, career expectations, professional identity, course design, support from others, and the campus environment in terms of learning motivation?”

The respondent codes are presented in the format of “serial number-grade-major.” The serial numbers are denoted as X1, 2, 3. 30; from the first year to the fourth year of study, they are, respectively, labeled as “01, 02, 03, 04”; the major is abbreviated using pinyin, such as calligraphy studies are denoted as “SF,” and accounting studies as “KJ.” In this study, the interview records of 26 out of 30 randomly selected respondents were used for coding analysis, factor extraction and model construction, while the interview records of the remaining 4 respondents were used for the test of theoretical saturation (see Table 1).

**Table 1:** List of 30 university students from Y University.

No.	Grade	Code	Major	Gender	Age
1	Junior	(X1-03-ZS)	Business administration	Male	20
2	Junior	(X2-03-ZF)	Calligraphy	Female	20
3	Junior	(X3-03-SZMTYS)	Digital media art	Female	21
4	Freshman	(X4-01-SJCDSJ)	Visual communication design	Female	18
5	Freshman	(X5-01-SF)	Calligraphy studies	Female	18
6	Sophomore	(X6-02-SF)	Calligraphy studies	Female	20
7	Freshman	(X7-01-XXJY)	Primary education	Male	19
8	Sophomore	(X8-02-SJCDSJ)	Visual communication design	Female	19
9	Freshman	(X9-01-SF)	Calligraphy studies	Male	18
10	Junior	(X10-03-SY)	Photography	Female	21
11	Sophomore	(X11-02-ZK)	CPA (certified public accountant)	Female	19
12	Freshman	(X12-01-GJ)	International education	Male	18
13	Junior	(X13-03-TXGC)	Communication engineering	Female	20
14	Freshman	(X14-01-ZNZZ)	Intelligent manufacturing	Female	19
15	Senior	(X15-04-DQ)	Electrical engineering	Male	21
16	Sophomore	(X16-02-SK)	Data science	Female	19
17	Junior	(X17-03-XXJY)	Primary education	Female	21
18	Junior	(X18-03-XXJY)	Primary education	Female	20
19	Sophomore	(X19-02-GJ)	International education	Female	20
20	Sophomore	(X20-02-GS)	Management	Male	19
21	Junior	(X21-03-ZK)	CPA (certified public accountant)	Female	21
22	Sophomore	(X22-02-ZK)	CPA (certified public accountant)	Female	20
23	Sophomore	(X23-02-ZK)	CPA (Certified Public Accountant)	Female	19
24	Sophomore	(X24-02-ZK)	CPA (Certified Public Accountant)	Female	19
25	Junior	(X25-03-XQJY)	Preschool education	Female	21
26	Senior	(X26-04-GJ)	International education	Male	22
27	Senior	(X27-04-SK)	Data science	Male	22
28	Senior	(X28-04-XXJY)	Primary education	Female	21
29	Senior	(X29-04-ZNZZ)	Intelligent manufacturing	Male	22
30	Freshman	(X30-01-TXGC)	Communication engineering	Male	18

The current grounded theory mainly consists of three schools: Glaser’s classic grounded theory, Strauss’s procedural grounded theory, and Camacho’s constructivist grounded theory. This study follows Strauss’s procedural grounded theory. Procedural grounded theory clearly divides the open coding, main axis coding, and selective coding into complete operational steps, allowing for a clear induction of initial concepts and sub-main categories from 100 original free nodes, ultimately refining the core category of “cognition-context dual drive.” The hierarchy is clear and traceability is strong, matching the research goal of this study to build a bottom-up motivation mechanism model from the bottom up. Procedural grounded theory strictly requires “interview collection-coding analysis synchronous iteration” and conducting theoretical sampling based on new categories, which precisely fits the two-stage sampling design of this study. Procedural grounded theory ultimately produces a theoretical model with causal, contextual, and mediating regulatory relationships, rather than purely abstract concepts. This study aims to clarify the multi-layer interactive path of learning motivation of undergraduate students in applied universities, and ultimately implement optimization countermeasures for courses, teachers, and resources. The clear transmission model generated by procedural grounded theory can directly connect with university education management practice, and is more in line with the dual research purpose of this study, “theory construction + practical implications.”

This study employed a simultaneous data collection + continuous comparison and iterative process to determine theoretical saturation. The coding was conducted by three researchers, QiaoxiaWang, Menglu Guo, and Linzhi He, in two rounds on 26 undergraduate students through semi-structured interviews. After each round of interviews, the coding was carried out, and the next round of interviews were adjusted simultaneously. If a certain category dimension was weak, corresponding characteristic respondents were targeted for supplementation. During the synchronous transcription and coding process, new interview texts were continuously compared with the existing coding categories, and analysis memos were written. The process tracked whether new concepts, sub-categories, or category relationships continued to emerge after each round of transcription. After completing the coding of 26 main samples, 4 additional theoretical sampling interviews were added for saturation verification. In the initial stage of the study, random sampling was not used as the selection criterion. Instead, purposeful stratified sampling was used to recruit the basic samples. The stratification dimensions included major categories (business and economics, engineering, media and communication, art), grade (freshman to senior), academic engagement level, and frequency of practical training participation. From all undergraduate students of Y application-oriented universities, 18 students covering all stratification characteristics were actively selected for the first round of interviews. The first round of 18 interviews was carried out simultaneously with the coding process. During the continuous comparison and memo writing process, 8 respondents were targeted for supplementation of interviews based on the newly emerged categories, dimensions, or ambiguous variable relationships that emerged from the coding. An iterative theoretical sampling was implemented: for example, when a new category “family planning and development guidance” was emerging, we would specifically recruit students involved in family business and those whose parents intervened in professional choices to supplement the interviews. After multiple rounds of theoretical sampling, 4 students who did not participate in the previous interviews were added as verification samples. The coding process was repeated in full to verify whether no new concepts, new associations, or new connections emerged, and to determine theoretical saturation, thus terminating the recruitment of all interviews.

This study set three saturation criteria: (1) No new core concepts or sub-categories were generated by the new data; (2) There were no new logical connections among the existing categories; (3) All dimensions and attributes of the categories were fully filled, with no missing descriptive information. After completing the coding of all 30 interviews, all three criteria were met, indicating that saturation of the theory was achieved. The recruitment of samples was terminated and no new respondents were added.

### 2.2 Data encoding and model construction

First is open coding. To conceptualize and categorize the original data and minimize subjectivity, in this study, open coding attempts to select the respondents’ original statements. Using the Nvivo15 software, meaningful segments were extracted from the original text sentence by sentence, processed through the “original expression → conceptualization → categorization” process, and redundant information unrelated to “learning motivation” was excluded. After repeatedly comparing, comparing, integrating and merging the overlapping and repetitive concepts in the original interview records, 36 initial concepts were obtained. Coding results: A total of 100 free nodes were extracted, merged into 36 initial concepts, and further integrated 17 variable categories (see Tables 2, 3).

**Table 2:** Example of the open coding section.

Some original sentences	Initial concept	Variable category
The AI era has been quite helpful to me. For instance, when I do not understand something, I just search it on Doubao.(X7-01-XXJY)	AI Facilitates Personal Development	AI-specific dimension
Now, we can use AI to summarize a lot of outlines and frameworks for us, which will help us learn better.(X23-02-ZK)		
The cost is lower than that of hiring financial staff. I think the use of artificial intelligence has caused a lot of anxiety.(X20-02-GS)	Anxiety about AI job replacement	
It’s not just about learning some basic things. The human mind itself is much more powerful and cannot be replaced by AI.(X23-02-ZK)	AI self efficacy	
The problems we encounter in practice are all unknown. Therefore, we still hope to have a little more hands-on experience.(X4-01-SJCDSJ)	High level of socialization desire	Professional understanding and expectations
Positive feedback is great. It gives me that sense of satisfaction.(X5-01-SF)	Positive feedback of internal drive	Self-efficacy, satisfaction, and motivation
Since I was in primary school, I have had a strong desire to become a teacher.(X5-01-SF)	Long-term career aspirations and academic advancement paths	Future career planning and employment requirements
Realizing such an excellent minister, and such a competent and responsible instructor, I am also very fortunate.(X6-02-SF)	Interacting with outstanding teachers and peers has effectively enhanced one’s cognitive abilities.	Peer influence
The parents hope that after I complete my studies in business administration, I can manage the family business.(X1-03-ZS)	Family planning and development guidance	Family influence
My high school teacher has also been constantly encouraging me.(X5-01-SF)	The encouragement and care from the teacher are very comforting.	Teacher influence
The laboratory computers are experiencing lag and data has been lost.(X11-02-ZK)	The hardware facilities need to be improved.	School resource conditions

**Table 3:** Categories formed through open coding.

Order number	Variable category (subcategory)	Initial concept/phrases (frequency)
1	AI-exclusive dimension (8)	AI Facilitating Personal Development (2), AI Enhancing Work Efficiency (3), AI Concerns About Job Replacement (2), AI Self-Efficacy (1)
2	Professional Understanding and Expectations (12)	Professional value recognition (5), strong socialization desire (4), prefer practical courses (3)
3	Self-efficacy, satisfaction, and intrinsic motivation (4)	Positive feedback of internal drive (2), sense of achievement brought about by improvement in performance (2)
4	Future Career Planning and Employment Requirements (9)	Long-term career aspirations and academic advancement paths (3), employment development and qualification accumulation (6)
5	Self-awareness (9)	Thinking ability development (2), social and practical growth (3), self-efficacy and growth confidence (4)
6	Internship environment (1)	The atmosphere during off-campus internship programs is oppressive. (1)
7	Peer influence (10)	Interacting with outstanding teachers and peers has effectively enhanced one’s cognitive level (6), group collaboration and mutual assistance in completing learning tasks together (2), and the exemplary role of class cadres has had a significant impact on personal development (2)
8	Scholarship factors (1)	Scholarships and other incentive measures have a positive guiding effect on learning behavior (1).
9	Learning pressure (1)	The intense learning pace imposes academic pressure (1)
10	School resource conditions (12)	Excessive class sizes (1);hardware facilities need improvement (3), class duration is prolonged (2), laboratory usage rights (2), course or teaching schedule arrangement (4)
11	Student behavior habits regulations (5)	Campus system learning and discipline (3), self-management of daily routines (2)
12	Family influence (5)	Family Planning and Development Guidance (3), Family Emotional Support and Deep Impact (2)
13	The Influence of Teachers (10)	The teacher’s encouragement and care are very comforting.(8); the negative impacts of school teachers are limited (2);
14	Class academic atmosphere (1)	The class atmosphere is excellent (1)
15	Autonomous time management (3)	Intensive course schedule (1), insufficient free time (2)
16	Course evaluation method (8)	Course assessment score structure (5), Classroom teaching organization form (3)
17	Out-of-school interpersonal relationships (1)	Interpersonal relationships are more complex in the workplace (1)

The second aspect is axial coding. Axial coding identifies and establishes relationships between categories based on open coding, reflecting the interconnections within the data. Through subjective clustering of 17 variable categories, this study ultimately identified the main categories (see Table 4), forming a logical chain among them: “external context → subjective cognition → motivational response.”

**Table 4:** The relationship construction of axial coding formation.

Fundamental category	Corresponding variable category	Domain conceptualization
An individual’s internal perceptions and expectations for personal growth	AI-exclusive dimension (8); Professional Understanding and Expectations (12); Self-Achievement Sense/Satisfaction/Intrinsic Motivation (4); Future Career Planning and Employment Requirements (9); Self-awareness (9)	Students’ perceptions, expectations, and intrinsic psychological motivations regarding their own abilities, professional fields, and future career development constitute the core internal drivers of personal learning and growth.
External Environment and Resource Support	Internship Environment (1), School Resources and Facilities (12), Course Evaluation Methods (8)	It refers to the objective material conditions, institutional frameworks, and external support systems available to individuals during their learning and practice processes, which directly influence the effectiveness of learning experiences and career preparation.
The impact of interpersonal relationships both on and off campus	Peer influence (10), Family influence (5), Teacher influence (10), Extracurricular interpersonal relationships (1)	It refers to interactions, value transmission, and behavioral influences from significant individuals within the family, school, and society, which shape an individual’s learning attitude and career choices.
Academic and behavioral constraints	Scholarship factors (1), academic pressure (1), constraints on students’ behavioral habits (5), Class Academic Atmosphere (1), self-management of time (3)	It refers to the rules, pressures, resource constraints, and time allocation faced by individuals in academic and daily life, reflecting the dynamic balance between external demands and personal autonomy.

The third is selective coding. Based on open coding and axial coding, this study further reorganized, integrated and analyzed the interview records. After abstracting the core categories, they were established as the influencing factors of learning motivation and their mechanisms, and a logical line was established as a clue: First, the individual’s internal cognitive system is the direct source of learning motivation. Second, the external context plays a role of awakening, regulating and stimulating the individual’s internal subjective cognition. It consists of external context conditions such as external environment and resource support, interpersonal and social influence, academic and life constraints. Core category establishment: Through the abstract integration of main categories, the core category is determined as “The ‘Internal Cognitive-External Context’ Dual-Driven Influence of Learning Motivation of Undergraduate Students in Applied Universities in the Digital Age.”

To clearly demonstrate the step-by-step coding logic of this research and enhance the traceability of qualitative analysis, the following provides a complete example of the five-level coding traceability chain, fully presenting the entire process of gradually refining from the original interview materials to the core theoretical category, and visually distinguishing the hierarchical progressive relationship among 100 free nodes, 36 initial concepts, and 17 variable categories:

Example 1 Original interview quote: “Because I have talked to relevant professionals and classmates, the digital AI agent will gradually standardize the employment rules for accounting industry practitioners. At the same time, it will replace some relatively basic tasks, using AI to replace human work, which is more efficient than manual accounting.” (X20-02-GS).

“And the cost is lower than hiring accounting staff. I think AI has caused a lot of anxiety, forcing us to work even harder.” (X20-02-GS).

Extracted initial code: AI improves work efficiency, AI causes career anxiety.

Subcategory (category): AI-specific dimension.

Main category: Personal internal cognition and growth expectations (core driving layer).

Core category: Cognition-Context-driven learning motivation generation core.

Example 2 Original interview words: “Now we can use AI to summarize a lot of outlines and frameworks for us, which enables us to learn better. I think currently, what AI has brought to me is positive, in terms of learning, it is positive.” “To do what AI cannot do, this is where we need to learn now, rather than merely focusing on learning some very basic things. Our own human thinking needs to be stronger, and it cannot be replaced by AI.” (X23-02-ZK).

Initial code: AI boosts personal development, AI improves work efficiency, AI enhances self-efficacy.

Subcategory (category): AI-specific dimension.

Main category: Personal internal cognition and growth expectations.

Core category: Cognition-Context-driven learning motivation generation core.

Example 3 Original interview words: “The computers in our laboratory are not very user-friendly. Sometimes when we first accessed that accounting information system, the computer in the classroom where we went was only usable in the first row, and the rest of us all crashed and could not access the system, which affected the progress of everyone’s learning.” (X11-02-ZK).

Extracted initial code: Hardware facilities need improvement.

Subcategory (category): School resource conditions.

Main category: External environment and resource support (basic guarantee layer).

Core category: Cognition-Context-driven learning motivation generation core.

Based on the above examples, we can clearly see that the original interview oral materials are decomposed into fragmented free nodes (initial code) → Semantic synonyms are merged to refine the initial concept, and then aggregated to form subcategories → Based on causal, contextual, and interactive logic, clusters are formed to obtain main categories → All main categories are integrated and summarized into the core category of this study, “Cognition-Context-driven Dual Drive.”

Fourth is model construction. In the digital-intelligent era, external contexts, including environmental and resource support, interpersonal and social influences, as well as academic and life constraints, impact students’ cognitive systems (professional awareness and expectations, self-achievement/satisfaction/motivation, future career planning, and employment requirements, self-awareness). Through the regulatory role of contextual fit, these factors ultimately drive the generation, reinforcement, or weakening of learning motivation. Specifically, personal internal cognition and development form the core driving layer; external environmental and resource support constitute the foundational support layer; interpersonal and social influences represent the relational influence layer; while academic pressure and behavioral constraints constitute the external constraint layer. These four layers interconnect horizontally: the internal driving chain determines the demand orientation of the foundational support chain, the external shaping chain adjusts the cognitive tendencies of the internal driving chain, and the dynamic equilibrium chain regulates the implementation efficiency of the former, collectively forming a closed-loop influence mechanism.

**Figure 1:**
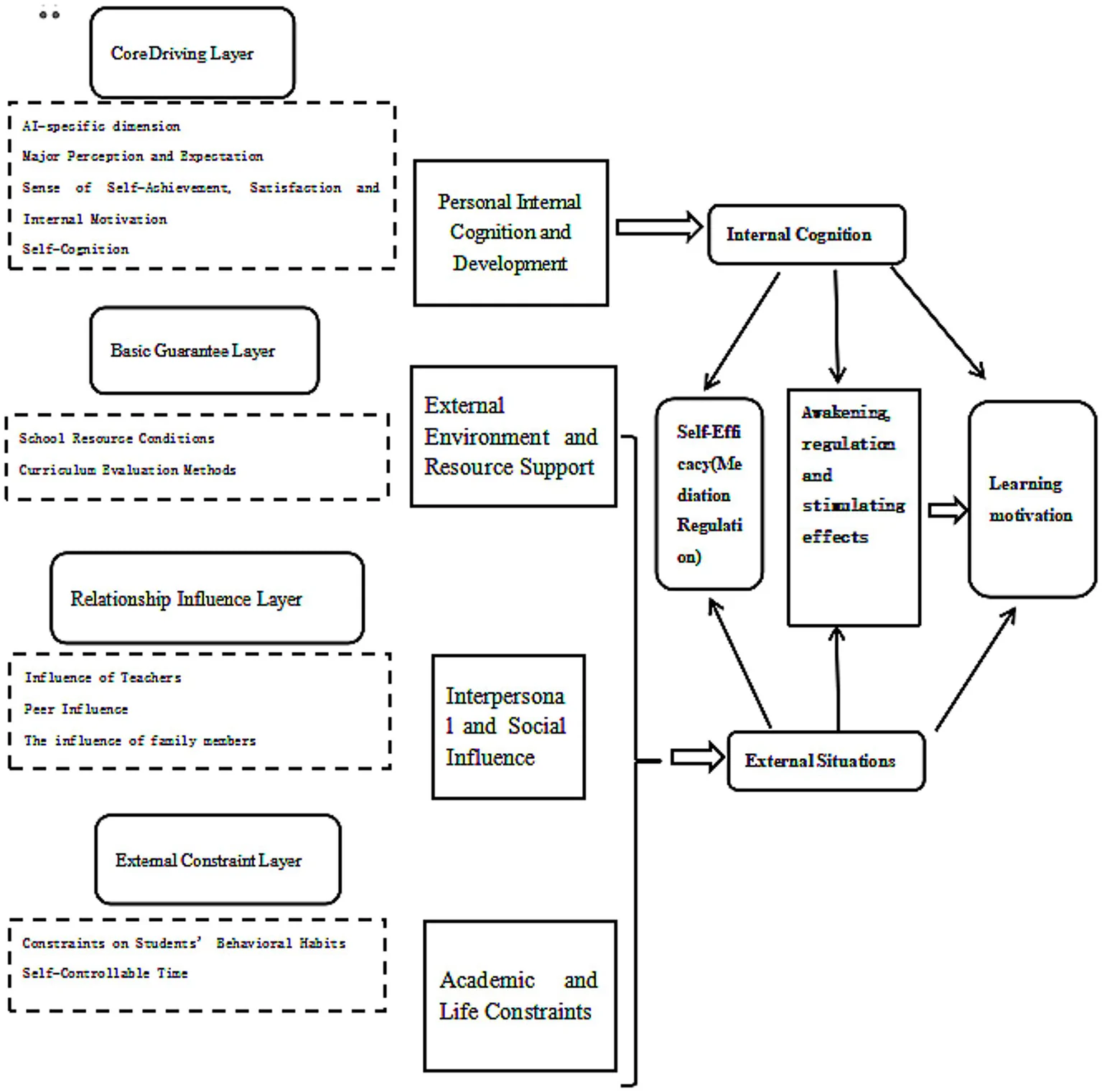
Model of the “Cognitive-Contextual” Dual-Driven Learning Motivation Formation Mechanism for undergraduate students in Digital Age-oriented applied universities. Self-efficacy is the core mediating variable of this model; arousal, regulation, and stimulation are the three-layer dynamic transmission mechanisms jointly generated by the interaction between internal cognition and external context.

Explanation: A few concepts that are supported by limited data (such as internship environment, off-campus interpersonal relationships, scholarship factors, study pressure, and class academic atmosphere) are no longer included in the model framework. They are only used as supplementary descriptive content to ensure that each dimension of the model has sufficient and diverse qualitative data for verification (see Figure 1).

### 2.3 Theoretical saturation test

Four interview texts that were not involved in the coding process were randomly selected and the same coding procedure was applied to them. No new concepts, categories, or association paths were identified, which proved the stability of the core categories and the model, and the saturation of the grounded theory sampling was achieved.

## 3 Characteristics of learning motivation among students at applied universities in the digital and intelligent era and the explanation of dual driving mechanisms

### 3.1 Characteristics of learning motivation manifestations

#### 3.1.1 Learning motivations have become more diverse

In the context of contemporary societal demands, the learning motivations of students at application-oriented universities have evolved beyond traditional, singular goals such as academic advancement or career preparation. They now exhibit a more diverse and reality-aligned trajectory, aligning educational values more closely with the pulse of real society (Wang, 2019). From rural teaching initiatives rooted in local communities to live-streaming support programs for agricultural development, to leading innovation transformations in family businesses, participating in social welfare volunteer services, and focusing on niche-sector entrepreneurship—all these activities demonstrate how student motivations are increasingly intertwined with societal needs and personal interests.

“Alternatively, I could leverage the internet to start live streaming and promote my hometown. My family comes from a rural area where we grow fruits. Through online platforms, I can showcase my hometown while contributing to rural revitalization and economic development. Our family owns ample land perfect for live streaming, making it particularly suitable for e-commerce through live broadcasts.” (X8-02-SJCDSJ).

One respondent explained: “I chose business administration as my major due to certain family circumstances. I received support from my family members, who owned a small business themselves, and they encouraged me to study this field to help expand and strengthen their family enterprise.”(X1-03-GSGL).

“I simply became a volunteer, stationed at our community entrance to register visitors from outside. While I did feel some fear, I found it profoundly meaningful. These experiences led me to consistently volunteer for classmates and organizations throughout my university years, in addition to participating in selection programs or the Western China Volunteer Program.” (X10-03-SY).

“I can clearly feel that this is not what I enjoy; that’s a step forward. I wanted to study acting and eventually become a comedian.”(X4-01-SJCDSJ).

This diverse range of learning motivations not only makes knowledge acquisition more targeted and practical but also fuels students’ intrinsic drive to explore proactively and apply what they learn, enabling them to realize their personal value while contributing vibrant energy to social development.

#### 3.1.2 Significantly influenced by external circumstances

The strength and manifestation of learning motivation are significantly influenced by factors such as the school environment, professional identity, and academic requirements (e.g., GPA), and personal engagement (e.g., research participation).

One respondent stated: “We hope the university can provide stronger support for students preparing for postgraduate entrance exams, including opening study rooms, extending closing hours, and ensuring a reliable water and electricity supply.” (X18-03-GJ).

“The computers in our lab aren’t very user-friendly. When we first tried using the accounting information system, only the computers in the front row of the classroom were functional; all the ones behind crashed and prevented us from accessing the system, significantly disrupting our learning progress.” (X11-02-ZK).

The strength of learning motivation is significantly influenced by external contexts. The infrastructure challenges and resource limitations students encounter in study rooms, computer labs, and other learning environments not only hinder academic performance but also underscore the necessity for schools to optimize learning environments and enhance supporting facilities.

### 3.2 Personal internal cognition and growth expectations: the Core driver of sustainable motivation

Self-efficacy is the core concept of Bandura’s social learning theory, referring to “an individual’s confidence in their ability to perform a specific behavior” (Bandura and Schunk, 1981).

#### 3.2.1 The dual guiding role of self-efficacy under AI cognitive differentiation in setting learning goals

The traditional theory holds that students with high self-efficacy are more likely to set challenging learning goals (such as taking the postgraduate entrance examination or publishing a paper), believing that they can achieve them through their efforts. In the context where AI has fully integrated into professional learning, students’ digital self-efficacy perception has further differentiated. The attitude towards the use of AI tools and the behavior of tool dependence have become the key mediators through which self-efficacy influences goal setting, resulting in two completely different goal orientations:

Type 1: Students with high self-efficacy, who can clearly distinguish the auxiliary value of AI from the core of autonomous learning, and have clear hierarchical development goals. These students possess a solid professional foundation and operational skills with digital tools, actively set long-term challenging goals such as pursuing higher education, obtaining certifications, or securing high-end job positions. They view AI as an auxiliary means to optimize learning efficiency, rely on their inner drive to deeply master core professional knowledge, and use AI to complete peripheral tasks such as data organization and visual assistance, without using AI as an excuse to avoid independent study.

One respondent said: “I think learning is like an exploration game. I find it very interesting. For instance, if I think something is important, it’s an internal drive of mine. I need to learn it, and then I might use the AI tool to help me improve the learning of this knowledge.” (X17-03-XXJY).

“Now we can use AI to summarize a lot of outlines and frameworks for us, which helps us learn better. I think for now, what AI has brought to me is positive, especially in terms of learning.” (X23-02-ZK).

“For me, in terms of the first stage’s goal, it’s about getting into university. But the overall goal is mainly to solve the employment problem. This employment issue actually includes things like starting a business, joining a regular organization, or entering a firm. For the study of accounting, this is the second stage’s goal.” (X20-02-GS).

“But if I think this thing is not important and does not have any impact on my future, then I might use AI to help me be lazy.” (X17-03-XXJY).

The second category: Students who are affected by AI substitution anxiety and have weak digital self-efficacy tend to have short-term and utilitarian goal planning, only setting basic employment and passing simple coursework as the minimum standards. Students in high-risk industries for AI substitution generally regard short-term survival through employment as their sole learning goal, lacking higher-level planning such as graduate entrance examination or skill improvement, and fearing that AI will squeeze the survival space of basic positions, thus having no time to deeply cultivate complex professional capabilities.

One of the respondents said: “What I really want is to get a good job position. I have already decided which position I will apply for.” (X3-03-SZMTYS).

“I think it’s very necessary for us college students to have internships and part-time jobs. We should start socializing as early as possible. Especially for those studying art, we cannot just focus on art alone; we definitely need to connect with society. Not to mention making a lot of money, you have to ensure your basic needs are met first. Only then can you pursue art.” (X5-01-SF).

When students are given sufficient autonomy in choosing their courses, they can independently select professional elective courses, academic practice projects, etc., based on their own interests, career plans and knowledge deficiencies. At the same time, they can flexibly arrange their own learning pace. In this way, they will gain a strong sense of subjectivity and control during the learning process. They will truly define learning as “their own business” rather than a passive task (Laur-Põder, 2023).

#### 3.2.2 The interactive mechanism between AI technology impact, self-efficacy, and students’ learning engagement

When facing difficulties (such as complex courses or the risk of failing), students with high self-efficacy are more likely to persist and will actively adjust their learning methods; while students with low self-efficacy may easily give up, weakening their learning motivation (Tang and Tseng, 2013).

Under the background of the digital and intelligent industry transformation, students’ perception of self-efficacy will be further differentiated by the employment impact brought by AI technology. AI-related career anxiety becomes a key intermediate variable that regulates self-efficacy and learning engagement. The first category: low self-efficacy → intensify AI substitution anxiety → reduce learning engagement. Some students majoring in digital media are aware that AI can efficiently complete repetitive tasks such as basic bookkeeping and 3D design. They are worried that their practical skills can be replaced by technology. Their digital self-efficacy is weak, which amplifies their employment anxiety and leads to behaviors such as avoiding practical training and procrastinating on assignments.

One respondent said: “In the digital age, it is very helpful for us students of digital media. For instance, interactive content, 3D AI can be produced for you immediately. However, in fact, it is very likely to be eliminated by this era. Therefore, many students do not want to attend classes anymore.” (X3-03-SZMTYS).

“I was already a bit lazy to begin with. Before the AI came along, most of the time I relied on my own brain to figure things out. But now that the AI is here, especially things like Doubao and Deepseek, I become more dependent on these tools, which causes my brain to think for a little longer on even the simplest tasks.” (X24-02-ZK).

“Instead of asking the AI directly how to solve it and getting a detailed explanation process, I’ll just compare it with the explanations and answers on the AI’s interface, and then think about the problem from my own perspective. Although following the AI’s thinking pattern can help solve the problem quickly, it’s not my own thinking process. So when encountering the next problem, I still will not be able to solve it.” (X24-02-ZK).

Type 2: High self-efficacy → Transform AI anxiety into learning motivation → Enhance engagement intensity. If students possess the ability to proficiently use professional AI tools and have sufficient confidence in their digital skills, the anxiety caused by the industry transformation brought by AI will, in turn, push them to deepen their expertise, actively increase the time spent on practical training, certifications, and skill practice, converting external pressure into long-term learning commitment.

One respondent said: “I might be able to achieve things that AI cannot. This is where we need to focus on learning now, rather than merely studying some very basic things. Our own human thinking needs to be stronger and cannot be replaced by AI.” (X23-02-ZK).

“Because I have talked to relevant professionals and classmates, the digital AI intelligent agent will gradually standardize the employment rules for accounting industry practitioners. At the same time, it will replace some relatively basic tasks, using AI to replace manual financial work, which is more efficient than human operation.” (X20-02-GS).

Moreover, the cost is lower than that of hiring financial staff. I think the use of artificial intelligence has caused a lot of anxiety, forcing us to work even harder. (X20-02-GS).

A comparison of the two groups reveals that AI anxiety does not have a direct positive/negative impact on learning behavior alone. Self-efficacy plays a core mediating role, determining whether students retreat and avoid or actively confront and deepen their focus on their chosen and preferred professional fields in the face of the pressure of AI industry transformation.

In addition, the inherent initiative brought by autonomous choice makes students more willing to invest their energy in delving into the knowledge domains they have chosen, like, and are willing to deepen: they have more patience when facing obscure theories and more motivation to persist when overcoming practical difficulties.

#### 3.2.3 Self-efficacy affects the sense of achievement in learning

High self-efficacy can reduce learning anxiety. Especially when combined with the use of AI tools, it makes learning more efficient, allowing students to enjoy the learning process more and thereby maintaining their intrinsic motivation. On the other hand, low self-efficacy is likely to trigger frustration, leading to a decline in learning motivation (Usher and Pajares, 2008).

One respondent said: “Because my major is an all-English accounting course, which requires translation. Without AI, I basically cannot understand what they are saying. So basically in every class, your mobile phone needs to be turned on with the translation function. I think first of all, AI definitely brings us positive benefits. It helps us learn a lot of knowledge.” (X23-02-ZK).

“I have been studying for 3 years now and I do not find it too difficult. During my internship, I felt that accounting work was quite friendly for me and I could accept it quite well. Before that, I had a certain resistance, but actually, it wasn’t that hard after getting started.” (X21-03-ZK).

Another shared: “Writing large calligraphic characters gave me a strong sense of achievement; positive feedback not only boosted my satisfaction but also stemmed from external incentives like completing assignments, pursuing awards, or competing with peers” (X5-01-SF).

Compared to passive learning approaches, voluntary learning behaviors maximize the activation of personal passion and aspirations, transforming learning from a tedious burden into a pathway for self-improvement and value realization, thus fostering more enduring and robust intrinsic motivation (Barrera et al., 2014).

### 3.3 External contextual system: a key guarantee for persistent motivation

#### 3.3.1 External environment and resource support

Ryan and Deci, (2017) argue that in educational contexts, “the granting of autonomy,” “positive feedback on learning outcomes,” and “support from peers and teachers” are key pathways for fulfilling three fundamental psychological needs: autonomy, competence, and belonging, and play a central role in enhancing learning motivation and effectiveness. High-quality external environments and resource support, such as well-equipped campus facilities, extensive professional literature databases, diverse internship and practical training platforms, and scientifically designed course evaluation systems, can remove objective barriers to individual learning exploration and establish a stable, reliable learning support framework (Wu, 2016).

One respondent remarked, “I hope the school will extend the opening hours of the calligraphy classroom. Preparing for postgraduate entrance exams requires extensive memorization of subjects like English and politics; we would appreciate having a dedicated study area for exam preparation to provide stronger support for students taking these exams. (X2-03-SF).

“In previous classes, there were probably just over 20 students. Our class now has 45 members, and the teacher may not be able to attend to everyone. Some students might genuinely want to learn but feel hesitant to approach the teacher; with so many students present, the teacher might not be able to see them all. “(X3-03-SZMTYS).

“We do not have enough power outlets in our school library; there’s only one smart area with both outlets and computers, but even that spot can sometimes be hard to secure.” (X8-02-SJCDSJ).

The study room can have a temporary storage area similar to that of a library; students can simply drop off their computers or tablets there. (X22-02-ZK).

When learners do not need to expend extra effort due to limited resources and can easily access knowledge tools, practical opportunities, and guidance, they develop a stronger sense of control and security during the learning process. This positive experience gradually transforms into intrinsic motivation for learning. Moreover, a favorable external environment often fosters a positive learning atmosphere that further stimulates learners’ curiosity and drive, encouraging them to devote more time and effort to acquiring knowledge and enhancing their skills.

#### 3.3.2 Interpersonal relationships and social impact

##### 3.3.2.1 The Influence of teachers

Teachers serve as central guides in college students’ academic and campus lives. Effective teacher-student interactions, tailored instructional guidance, and humanistic care help students develop a sense of psychological belonging on campus, optimize their learning mindset, and cultivate sustained positive motivation for learning (Niemiec and Ryan, 2009). This sense of belonging not only alleviates the loneliness and confusion associated with studying independently but also transforms into an intrinsic drive to actively participate in classroom discussions, engage in research practices, and explore cutting-edge areas of their discipline. Furthermore, the intellectual exchanges, experience sharing, and positive feedback generated through constructive interactions continuously reinforce a sense of achievement and enjoyment in learning, significantly enhancing interest in professional knowledge exploration and fostering a virtuous cycle of “interaction—belonging—active participation—interest enhancement” (Radovan and Makovec, 2015).

One respondent shared: “At first, I was somewhat concerned about accounting because I wasn’t good at math. Then, during my freshman year, I took Principles of Accounting taught by Professor Duan. She explained the subject very well, which made me realize accounting could be quite interesting, and from then on, I no longer feared it.”(X21-03-ZK).

“I believe teachers have a significant impact on students’ aesthetic sensibilities. We currently have three calligraphy classes that teach the same content, but each has different instructors. Students in other classes produce work that feels distinctly different from mine in terms of style. Our teachers may prefer a more regular, straight-line approach, whereas our class tends to adopt a more square and precise style overall.” (X5-01-SF).

The teaching style and professional instructional competence of educators directly determine students’ interest in specialized learning and help dispel stereotypical negative perceptions about the discipline. Moreover, teachers’ personal teaching approaches, aesthetic preferences, and behavioral principles subtly shape students’ learning styles and individual habits.

##### 3.3.2.2 Peer influence

Companionship encompasses role models among immediate family members, senior student leaders, classmates, and members of various campus clubs. The behaviors, learning attitudes, and growth trajectories of peer groups profoundly influence college students’ learning motivation and personal development across four dimensions: goal setting, personality formation, problem-solving skills, and friendship selection.

“Because my sister was truly an exceptional student. She earned her bachelor’s degree from Hunan University and was directly admitted to Zhejiang University for graduate studies. Observing her journey, I realized that I too needed to attend university someday. I could not continue living passively like in middle school; doing so would inevitably leave me without access to education. (X21-03-ZK).

“I’m currently the Head of Public Relations for the College Student Union. Our president has had a profound influence on me. He mentored me from my freshman year as a student council member and personally guided me in tasks like video editing, graphic design, and illustration. He’s truly exceptional.” (X8-02-SJCDSJ).

“In my freshman year, I first ran for a position on the class committee and then joined the Student Union, which proved quite beneficial to me. Being relatively introverted before, increased interactions with senior students and teachers helped me become more confident. Through the Student Union, I also met many outstanding faculty members and seniors, broadening my perspective and teaching me how to handle tasks and communicate effectively.”(X6-02-SF).

“Professor Duan, our dedicated mentor in the professional teacher program, has had a profound impact on me. He always plays jokes with us while simultaneously guiding us on how to study our major, helping us develop plans, and setting clear directions for our development. (X6-02-SF).

“My psychology teacher was truly wonderful. He was the first person after my parents to care about whether I felt cold in winter, if I was well-fed, and so on. I was deeply moved at that time.” (X6-02-SF).

“I also feel that my classmates have had a significant impact on me. Those who just go through the motions day by day can still influence you if you spend too much time with them. Around me, there are many ambitious individuals; I prefer spending time with more motivated classmates who share similar goals and aspirations—I truly enjoy being around such people.” (X5-01-SF).

Through modeling behavior, imparting skills, and providing emotional support, peers significantly influence college students’ learning attitudes, growth plans, and personality development, highlighting the crucial role of peer relationships and positive interpersonal support in student development.

##### 3.3.2.3 Impact of social interpersonal relationships

Campus interpersonal relationships serve as a microcosm of broader social interactions, while off-campus internships and other social practices allow students to gain early exposure to real-world workplace environments. Such social experiences and networking opportunities exert both positive reinforcement and negative impacts on college students’ learning motivation, career perception, and social attitudes, with the latter primarily manifesting in two aspects: impaired career confidence and social anxiety.

One respondent remarked: “During my internship, I filled out all documents with extreme care. Any mistake would render them completely invalid. At work, I felt I had to remain constantly vigilant and never let my focus waver, which made me realize I would never want to work for such a state-owned enterprise. (X21-03-ZK).

“I think school is essentially a microcosm of society. How you interact with classmates and teachers—these are just examples; in the workplace, things can become even more complex: how to deal with colleagues or supervisors. For me, it’s quite challenging; I do not know how to navigate these interpersonal dynamics.”(X21-03-ZK).

Social relationships within campus and workplace environments exert both positive empowering effects and negative impacts on college students’ learning motivation, career perception, and social mindset. Additionally, internship pressures and anxiety about complex social interactions can influence their career choices and learning attitudes.

#### 3.3.3 Academic pressure and behavioral constraints

Moderate academic and lifestyle constraints, such as reasonable course evaluation standards, fostering a positive learning environment in the classroom, and scholarship incentive mechanisms based on academic performance, establish clear behavioral boundaries and goal-oriented frameworks for individual learning, helping learners develop consistent study routines and self-discipline. These controlled constraints transform into positive external motivation, encouraging learners to manage their time autonomously, avoid inefficient learning behaviors, and accumulate a sense of achievement through achieving phased goals, thereby reinforcing intrinsic learning motivation (Seli and Dembo, 2020). However, excessive constraints, such as overwhelming academic pressure, rigid behavioral norms, or severely limited autonomous time—disrupt the balance between external demands and personal autonomy. Such conditions not only diminish learners’ curiosity and enthusiasm but may also trigger resistance and burnout, ultimately weakening learning motivation and leading to a vicious cycle of “high-pressure constraints → passive compliance → motivational exhaustion” (Vansteenkiste et al., 2006).

One respondent remarked: “We’re required to submit practical training reports for all hands-on courses, yet each report typically involves at least five experiments—one for each experiment and totaling only a few thousand words. However, our course schedule is extremely packed, leaving little time; therefore, we are finding alternative ways to assess learning outcomes instead of relying on these reports.”(X23-02-ZK).

“Can we reduce the use of flipped classrooms a bit? For example, in courses like Marxist Theory and Military Theory, they frequently employ this approach. These are subjects we study alongside other requirements. We originally needed to pass the CET-4 exam, but our preparation time was likely insufficient. Moreover, the flipped classroom requires us to create PPTs and organize teams to gather materials, among other tasks. The tight schedule makes us feel overwhelmed by academic pressure.” (X24-02-ZK).

“The lack of transparency regarding regular performance evaluations has caused psychological distress among some students, as these grades are determined solely by teachers and are not visible to us. We request that instructors for each course provide us with the regular evaluation records; any discrepancies can then be raised promptly, thereby ensuring exam fairness.” (X25-03-ZK).

Moderate academic constraints, such as well-designed assessments and positive incentives, can help define learning boundaries for college students and strengthen their intrinsic motivation. However, excessive academic pressure and rigid behavioral norms (e.g., excessive reporting requirements, or frequent flipped-classroom assignments—disrupt autonomy balance, foster resistance, and undermine learning motivation.

## 4 Discussion

Based on the motivation influencing mechanism derived from the grounded analysis of students in applied universities in this study, this section will compare and contrast the research results with existing domestic and international related literature to further explain the theoretical value and academic contributions of this study.

First, the conclusion of this study forms a complement to the existing research on college students’ learning motivation in China. Li et al. (Li et al., 2024) believe that learning motivation is stable and diversified, including internal (such as learning interest, ability pursuit) and external (such as honor acquisition, altruistic orientation) driving forces, and avoiding single-purpose utilitarian driving forces is crucial for alleviating “internal competition” and cultivating comprehensive talents. Stable and diversified learning motivation is the key to the comprehensive implementation of educational policies and the comprehensive cultivation of talents, which can promote multi-dimensional positive performance competition and lifelong comprehensive development of college students, avoiding single-purpose utilitarian learning driving forces, and better matching the needs of comprehensive talent cultivation, which is an effective way to alleviate the “internal competition” predicament of college students. Lu Kecheng and Mao Jinping’s earlier research pointed out that college students’ learning motivation has the characteristics of diversity, utilitarianism, and instability, but the early research did not take into account the background of the AI digital era and did not distinguish the differences between public and applied university groups. This study found that the utilitarian motivation of students in applied universities is simultaneously overlaid with AI career anxiety, and the motivation diversification is manifested in characteristic directions such as rural revitalization, family business management, and innovation and entrepreneurship, expanding the research boundaries of traditional college student motivation types.

Second, this study extends the applicable scenarios of Bandura’s self-efficacy theory and Ryan and Deci’s self-determination theory. The existing self-determination theory focuses on three psychological needs of autonomy, competence, and belonging, and Yang et al. (Yang et al., 2024) believe that enhancing self-efficacy, stimulating autonomous motivation, and developing positive and proactive rumination thinking are effective strategies to intervene in college students’ sports learning engagement, which has practical significance for improving the level of college students’ sports learning engagement. Zheng, (2023) believes that the popularization of higher education brings about the diversity of students’ psychological states, and the different resource allocation situations of different types of universities lead to significant differences in competition rules, and should recognize the potential positive effects of external learning motivation and deeply consider the role of “individual-environment matching” in improving the effectiveness of extracurricular activities. Peng, (2023) found that the different meanings of learning objects for students’ subjects is the fundamental reason for the differences in learning engagement among students with different academic achievements; the students’ perception of the learning context, learning motivation, emotional investment, cognitive investment, behavioral investment, and learning gains constitute a mutually influencing and restrictive learning engagement mechanism, and learning interest, academic resilience, and autonomous learning are the key emotional, cognitive, and behavioral attributes for students to achieve high academic achievements. Xue (2023) found that online learning motivation and self-regulated learning are two important factors affecting the effectiveness of online teaching; the motivation at the language level, learner level, and learning context level is strong; the students’ self-regulated learning ability is at an upper-middle level. This study combines the digital education scenario and adds two contextual variables of AI tool usage cognition and digital resource fairness to explain how the digital environment regulates students’ sense of competence and internal motivation; at the same time, in response to the resource shortcomings of applied universities, it improves the inhibitory path of external environmental constraints on self-efficacy and enriches the application of social cognition theory in private higher education.

Third, most existing research on digital education focuses on the technological empowerment effect of public universities, and pays less attention to the digital learning predicament of students in applied universities. This study confirms that the insufficient digital facilities and scarce teachers in applied universities will significantly weaken students’ learning motivation, and this conclusion compensates for the existing research’s neglect of the applied university group. Overall, based on the “cognition-context” dual-drive model derived from grounded theory, this study not only confirmed the core viewpoints of the classic motivation theory, but also expanded it by integrating the unique research scenarios of the digital age and application-oriented universities, thereby forming a localized analytical framework suitable for this group.

## 5 Relevant recommendations

### 5.1 Recommendations for supporting the diversification of learning motivations among college students in the digital and intelligent era

Internal factors constitute the fundamental drivers of students’ learning motivation, with diversified shifts in learning motivations and exploration of personal meaning being particularly crucial. Currently, students’ learning motivations have transcended the traditional single focus on academic advancement or employment, manifesting in diverse forms such as taking over family businesses, engaging in rural teaching programs, or providing live-streamed agricultural support. This shift essentially represents students’ proactive exploration of pathways for self-actualization, aligning their learning goals more closely with personal needs and social realities. Exploring the meaning of one’s existence represents a deeper level of internal motivation: when students connect learning with core questions like “What kind of person do I want to become?” or “What value can my learning bring?,” they develop intrinsic aspirations that transcend utilitarian considerations and motivations that prove more enduring and stable than external incentives (Frankl, 1966). However, under the impact of digital and intelligent technologies, the anxiety of AI replacing human jobs will intensify the utilitarian and short-sighted mentality of students. Many students only pursue the basic employment requirements and abandon long-term plans for skill improvement, resulting in a reduction of their internal development motivation.

Recommendations: The first family needs to transform the traditional utilitarian educational concept, abandon the single evaluation orientation of “only focusing on academic success and employment,” and actively communicate with their children about the industry AI transformation trends, objectively explain that AI can only replace repetitive basic work, while high-end comprehensive professional capabilities and innovative thinking cannot be replaced by artificial intelligence, thereby alleviating their children’s panic about AI jobs. They should respect students’ diverse career choices, not forcibly limit the employment paths of easily impacted majors such as finance and economics, and embrace diverse development directions such as live-streaming for agricultural assistance, creative arts, and innovation in family businesses. They should set an example by sharing lifelong digital learning experiences, guiding students to establish a rational understanding that “AI is a tool rather than a career threat,” and reducing extreme AI anxiety that leads to learning slackness and short-term coping behaviors. The second school should offer digital career value general education courses to alleviate AI anxiety and cultivate positive AI self-efficacy. Specialized lectures on “Professional Career Development in the Digital Age” should be added, and the application boundaries of AI in various industries should be dissected by major: for finance majors, intelligent tax accounting only replaces bookkeeping and accounting, while high-end risk control, auditing, and management accounting still rely on human comprehensive judgment; for media and communication majors, AI drawing and modeling are only auxiliary creations, while original creativity and aesthetic planning are core competitiveness; for liberal arts and traditional engineering majors, they should focus on explaining the emerging positions brought by industry digitalization upgrades, dispelling students’ one-sided perception that “the profession is eliminated by AI”; the courses should be accompanied by practical AI skills experiences, allowing students to operate professional AI tools themselves, improving digital operation proficiency, and solidifying AI self-efficacy, converting AI anxiety into the motivation for skill improvement. The third teachers should conduct personalized AI psychological counseling for students with AI efficacy polarization: for students with weak digital operation skills and fear of AI, one-on-one simple AI tool teaching should be carried out to lower the threshold of digital learning; for students deeply trapped in AI substitution anxiety and with short-term learning goals, combined with real industry recruitment cases, the ability requirements for composite talents in enterprise recruitment should be sorted out, guiding students to set long-term and high-level professional development goals, balancing the utilitarian learning mentality under the impact of AI.

### 5.2 Recommendations regarding condition support and interpersonal relationships

External factors serve as crucial supports for activating and sustaining students’ learning motivation, with school infrastructure, educators, and peer interactions forming the core external influence framework. High-quality educational resources, including well-equipped laboratories, extensive literature databases, and diverse practical platforms—eliminate objective barriers to learning while providing stable support, enabling students to enhance their sense of control over learning through accessible resources and thereby stimulating their curiosity. Teachers’ professional competence, instructional approaches, and mentoring attitudes directly shape students’ learning experiences; targeted academic guidance and positive feedback effectively boost learning confidence, while engaging teaching methods increase learning enjoyment. Peer interaction is equally vital: strong peer relationships and a positive learning environment create positive reinforcement effects, whereas collaborative group work and mutual assistance not only foster a sense of belonging but also stimulate learning initiative through intellectual exchange. Conversely, issues such as inadequate resource availability, insufficient teacher guidance, or negative peer influences can significantly undermine students’ learning motivation and even lead to academic burnout (Schaufeli et al., 2002).

Recommendations: First, implement a hierarchical digitalization of campus hardware to reduce the differences in AI usage among different majors. Based on the differentiated digitalization needs of each major, configure training resources: for finance and accounting, digital media, and big data majors, equip with intelligent tax systems, AI design, and data analysis tool rooms, extend the opening hours of AI training classrooms; for majors with less AI application, such as calligraphy, traditional liberal arts, and electrical engineering, provide lightweight AI auxiliary tools (text analysis, intelligent drawing, literature review software), complete the basic digital learning conditions, and eliminate the AI resource gap between different majors; uniformly build a campus cloud AI learning platform, allowing all students of all majors to use professional AI tools for free, balance the opportunities for students of different majors to access digital resources, and weaken the AI self-efficacy stratification caused by equipment differences.

Second, conduct specialized training for teachers’ digital teaching capabilities, strengthen AI teaching guidance, enhance teachers’ professional capabilities and teaching innovation awareness, adopt diversified and interactive teaching methods to avoid the traditional single teaching mode; at the same time, strengthen the guidance responsibilities of teachers, communicate and exchange with students regularly, provide academic support and psychological counseling in a timely manner, and motivate students’ learning enthusiasm through positive feedback (Tas, 2016). In terms of peer interaction, the school and teachers should actively build a peer collaboration platform, organize group learning, topic discussions, and practical mutual assistance activities, guide students to establish a positive peer relationship, create a positive and upward learning atmosphere in the class and campus, and let peer influence become a positive force to strengthen learning motivation (Winga et al., 2016). Regularly organize AI teaching workshops by major: finance teachers train on the teaching methods of intelligent tax AI training, art teachers learn the classroom design of AI-assisted creation, liberal arts teachers master the teaching norms of text AI tools; the training content includes two core modules: one is how to use AI tools to simplify class knowledge organization, expand cases, and enhance class interest; the second is how to guide students to use AI reasonably, distinguish between tool assistance and total plagiarism, and promptly correct students’ excessive reliance on AI and giving up independent thinking learning habits; require teachers to regularly demonstrate the practical operation of professional AI tools in class, through positive teaching demonstrations to enhance the AI usage confidence of all students.

Third, build a positive peer AI mutual assistance atmosphere to eliminate the anxiety of digital peer comparison. Form AI skills mutual assistance groups by class and major, encourage students who are proficient in digital tools to share operation skills, and assist students with weak digital foundation; guide students to rationally view the peer AI skill gap, clarify that AI is only an auxiliary means to improve efficiency, professional core thinking and practical ability are the core competitiveness for employment, reduce the inferiority complex and avoidance of learning psychology caused by the digital ability gap among peers.

Fourth, formulate a unified AI tool usage norms for the entire school, improve the AI teaching assessment system. Develop the “Guidelines for Undergraduate Professional Learning of AI Tools,” clarify the annotation norms for AI content in assignments, training reports, and course papers, require students to clearly distinguish between original content and AI-assisted generated content; reconstruct the course evaluation system, incorporate “reasonable use of AI assistance + independent original achievements” into the training score standard, reduce the score for pure written recitation, increase the weight of independent practical operation, original design, and independent analysis; set a deduction mechanism for assignments that copy AI results and have no independent thinking, from the institutional level to restrain the unrestrained abuse of AI.

### 5.3 Recommendations for practical teaching scenarios

With the evolution of educational philosophies and shifting societal demands, the traditional single-dimensional classroom teaching model has gradually declined, while students’ learning motivations have become more aligned with social practice and exhibit greater vitality. Conventional classrooms predominantly focus on theoretical instruction, lacking connections to real-world contexts, which often leads students to perceive learning content as “disconnected from reality” and consequently weakens their motivation (Mensah and Osei, 2025). In contrast, learning scenarios closely tied to social practice, such as internships, field research, corporate collaboration projects, and live-streaming initiatives supporting agriculture, allow students to directly appreciate the practical value of knowledge, integrating learning with solving real-world problems and addressing societal needs. Such immersive experiences effectively stimulate students’ curiosity and initiative, fostering more enduring learning motivation. This transformation fundamentally represents a shift in learning orientation, from “learning for knowledge acquisition” to “learning for practical application,” aligning with the core requirements of cultivating applied talents. However, challenges such as an incomplete practical teaching framework and poor integration between classroom instruction and hands-on practice hinder the full realization of practice-oriented learning motivation. In the era of digital intelligence, practical teaching must integrate the logic of human-machine collaboration, balance the efficiency of AI tools with students’ independent practical skills, and avoid the loss of autonomous competence caused by excessive reliance on AI.

Recommendations: First, universities should accelerate the reform of teaching models by reducing the dominance of traditional single-classroom instruction and establishing an integrated “classroom and practice” education system. This involves incorporating social practices and industry needs into curriculum design, increasing the proportion of practical courses, and enabling students to learn through hands-on experience and improve through exploration. Second, strengthen university-industry and university-local collaborations to establish stable off-campus practice platforms that provide students with diverse opportunities for internships, research projects, and volunteer services, Enable students to immerse themselves in real social scenarios, experience the practical value of knowledge, and strengthen their motivation for learning through practical orientation. Collaborate with industry partners to introduce real commercial AI systems, allowing students to directly align their on-campus training with the digital standards of the workplace; enterprise mentors will simultaneously explain the usage boundaries of industry AI tools and inform students how enterprises distinguish AI-assisted content from employees’ original capabilities, helping students establish a rational AI usage cognition that is in line with the workplace, and preventing them from developing bad learning habits of relying entirely on AI during their school years. Third, expedite faculty transformation by developing a team of dual-qualified teachers who can guide students in applying theoretical knowledge to solve practical problems, helping them achieve a sense of accomplishment through practice and further stimulating intrinsic learning motivation. Fourth, improve the practical teaching evaluation system by incorporating practical performance and problem-solving skills into assessment criteria, encouraging students to prioritize practical learning and actively participate in diverse hands-on activities to continuously reinforce their learning motivation. The assessment of practical outcomes no longer solely relies on the completeness of the final product. An additional scoring item, “Record of the Autonomous Thinking Process,” has been added. Students are required to submit complete materials documenting their AI usage ideas, self-corrections, and independent practical operations. Even if students complete the basic steps with the help of AI, if they lack independent analysis and original optimization steps, their training scores will be significantly reduced. This evaluation approach forces students to reduce their excessive reliance on AI tools.

## Data Availability

The original contributions presented in the study are included in the article/supplementary material, further inquiries can be directed to the corresponding author.
